# The regulator HlyU, the repeat-in-toxin gene *rtxA1*, and their roles in the pathogenesis of *Vibrio vulnificus* infections

**DOI:** 10.1002/mbo3.48

**Published:** 2012-11-28

**Authors:** Moqing Liu, Jorge H Crosa

**Affiliations:** Department of Molecular Microbiology and Immunology, Oregon Health and Science UniversityPortland, Oregon

**Keywords:** Gene regulation, HlyU, pathogenesis, repeat-in-toxin, *Vibrio*

## Abstract

HlyU is a master regulator that plays an essential role in the virulence of the human pathogen *Vibrio vulnificus*. One of the most noteworthy characteristics of HlyU regulation in this organism is its positive control of the expression of the repeat-in-toxin (RtxA1) gene, one of the most important virulence factors accounting for the fulminating and damaging nature of *V. vulnificus* infections. In this work, we reviewed the latest studies of RtxA1 in this bacterium and highlight the mechanism of gene regulation of *rtxA1* expression by HlyU under a broader gene regulatory network.

## Introduction: Infection and Virulence

*Vibrio vulnificus* is an opportunistic human pathogen that causes fatal primary septicemia or necrotizing wound infections. Septicemia usually occurs in patients who are immunocompromised, suffering from hemochromatosis, or with underlying liver disorders such as alcoholic cirrhosis or other liver disease (Vollberg and Herrera 1997; Haq and Dayal 2005; Vinh et al. 2006; Muldrew et al. 2007). In most of these patients, iron is present at higher than physiological level (Bullen et al. 1991; Hor et al. 1999). However, otherwise healthy people can also experience severe wound infections, which progress rapidly to cellulitis, ecchymosis, and necrotizing fasciitis at the site of infection, and could even lead to sepsis and death (Fujioka et al. 2003; Oliver 2005; Mouzopoulos et al. 2008; Jones and Oliver 2009).

During *V. vulnificus* infection, the bacterium reaches the intestine and then invades the bloodstream by crossing the intestinal mucosal barrier of the host, which results in the systemic invasion noted by the extensive tissue damage that occurs after ingestion of contaminated seafood (Kim et al. 2008; Lee et al. 2008a; Lo et al. 2011). *Vibrio vulnificus* damages macrophages during the early phase of infection and causes apoptosis of macrophages in vivo (Kashimoto et al. 2003; Tsuchiya et al. 2007). The bacterium replicates extensively in the interstitial fluid, and then causes damage, with the secretion of a large array of extracellular factors including capsular polysaccharides (Wright et al. 1990; Smith and Siebeling 2003), siderophores (Helms et al. 1984; Litwin et al. 1996), cytolytic hemolysin (Gray and Kreger 1987; Kim et al. 1993), protease (Wang et al. 2008; Shinoda and Miyoshi 2011), phospholipase A (Koo et al. 2007), and repeats-in-toxin (RtxA1) (Lee et al. 2007a; Liu et al. 2007; Kim et al. 2008). Flagella and pili also play important roles in the virulence phenotype of *V. vulnificus* (Kim and Rhee 2003; Lee et al. 2004a; Paranjpye and Strom 2005). In this review, we will focus on one of the most important virulence factors of *V. vulnificus*, the RtxA1 toxin. We will discuss the pathogenic role of RtxA1 as well as the regulation mechanism of this toxin by a regulator protein, HlyU.

## The Repeat-in-Toxin (RtxA1) of *V. vulnificus*

### The role of RtxA1 in the pathogenesis of *V. vulnificus* infection

Numerous secreted virulence factors have been proposed to account for the fulminating and destructive nature of *V. vulnificus* infections. The repeat-in-toxin RtxA1 that is related to the multifunctional autoprocessing RTX toxin (MARTX) of *V. cholerae* is one of the most important virulence factors in *V. vulnificus*. This 15,618 bp gene-encoding RtxA1 is the largest open reading frame of the *V. vulnificus* genome, and RtxA1 is recognized as the largest single-polypeptide toxin in *Vibrio* species. There are other two homologs of the *rtxA1* gene identified in *V. vulnificus*; however, none of their mutants exhibit a difference in virulence compared with the wild type (Liu et al. 2007; Kim et al. 2008).

So far, several groups have tested the virulence of the *rtxA1* mutants using either the iron-overloaded or the iron-normal mouse models. The LD_50_ varies based on the bacterial strain, mouse species, and the infection route. Table 1 summarizes the results from different research groups. Overall, the ratio of the LD_50_-*rtxA1* mutant/LD_50_-wild type varies between ∼50 and 2650. Basically, the LD_50_ fold change is higher in the iron-overloaded mouse model. One study suggested that there was no obvious difference between the LD_50_ of i.p. and i.g. infection routes (Kim et al. 2008). However, in a different study it was shown that the i.g. route is the more effective infection approach (Kwak et al. 2011). This discrepancy could be due to the different mouse species used by these two groups, and further investigation is needed.

**Table 1:** The virulence results for various *rtxA1* mutants from different groups

	LD_50_					
					
*Vibrio vulnificus* strain	WT	*rtxA1* mutant	Fold change MT/WT	Mouse species	Infection route	Reference
MO6-24/O	5.87	2.68 × 10^3^	456	Iron-overloaded ICR mice	i.p.	Lee et al. (2007a)
CMCP6	5	2.5 × 10^3^	500	Iron-overloaded CD1 mice	i.p.	Liu et al. (2007)
MO6-24/O	5.5 × 10^5^	5.5 × 10^7^	100	Iron-normal CD1 mice	i.p.	Kim et al. (2008)
MO6-24/O	8.0 × 10^5^	7.0 × 10^7^	88	Iron-normal CD1 mice	i.g.	Kim et al. (2008)
MO6-24/O	1.8 × 10^5^	3.2 × 10^7^	178	Iron-normal C57BL/6 mice	i.g.	Kwak et al. (2011)
CMCP6	2.4 × 10^4^	6.3 × 10^7^	2625	Iron-normal C57BL/6 mice	i.g.	Kwak et al. (2011)
CMCP6	7.3 × 10^5^	1.0 × 10^8^	137	Iron-normal C3H/HeN	i.p.	Lo et al. (2011)
CMCP6	3.9 × 10^4^	1.9 × 10^6^	49	Iron-normal C3H/HeN	i.v.	Lo et al. (2011)
CMCP6	1.1 × 10^5^	8 × 10^6^	73	Iron-normal C3H/HeN	s.c.	Lo et al. (2011)

i.p., intraperitoneal injection; s.c., subcutaneous injection; i.v., intravenous injection; i.g., intragastric route.

It has been suggested that the contact with host cells is a prerequisite to the acute cytotoxicity of *V. vulnificus* and the expression of the *rtxA1* toxin gene increased in a time-dependent manner after the host cell contact (Kim et al. 2008). When *V. vulnificus* infected HeLa cells, RtxA1 colocalized with actin and caused actin aggregation that led to a significant decrease in the F/G actin ratio. The RtxA1 toxin induced cytoskeletal rearrangements and plasma membrane blebs, thus resulting in necrotic cell death (Kim et al. 2008). This group also reported that like other repeat-in-toxin toxins, RtxA1 caused hemolysis of human RBC by forming pores at the cell membrane, while the PEG protection assay suggested that the estimate pore radius was approximately 1.63 nm (Kim et al. 2008). In another study carried out in CMT-93 murine intestinal epithelial cell and in a Caco2 human intestinal cell line, Chung et al. (2010) suggested that the generation of host cellular reactive oxygen species (ROS) via Rac2 cooperative Nox1 activation in response to *V. vulnificus* RtxA1 is a major contributor to the necrotic cell death syndrome. However, when the INT-407 human intestinal epithelial cell line was used, the study from Lee et al. (2008a) suggested that RtxA1 induced apoptotic death through a mitochondria-dependent pathway, and these apoptotic processes could also be induced by the bacterial supernatant. The discrepancy between necrotic and apoptotic cell death induced by *V. vulnificus* is likely related to the different eukaryotic cell type used, infection time, or the number of bacteria used. Recently, Lo et al. characterized an RtxA1-deficient mutant that showed a ∼2-log reduction in virulence for mice when introduced by various routes (Lo et al. 2011). Compared with the wild type, the *rtxA1* mutant was impaired in survival at the infection site and in spreading into the bloodstream, and was more readily cleared from the macrophage-rich mouse peritoneal cavity and phagocytosed by murine macrophages; thus, the RtxA1 of *V. vulnificus* appears to be important for bacterial survival by protecting the organism from phagocytosis. Coincidently, Kim et al. (2008) reported that an *rtxA1* deletion mutant was defective in invading the bloodstream in ligated ileal loops of CD1 mice. More recently, Jeong and Satchell (2012) demonstrated that RtxA1 is essential during the early stages of invasion to promote the initiation of the infection and dissemination to the bloodstream. Using histopathological approaches, this group also found that RtxA1 caused villi disruption, epithelial necrosis, and inflammation of the mouse small intestine (Jeong and Satchell 2012). Overall, taking together the results described in this section it is obvious that the RtxA1 toxin is multifunctional and plays an essential role in the pathogenesis of *V. vulnificus* infections.

### The secretion of the RtxA1 toxin

The secretion of RTX toxin by type 1 secretion system (T1SS) is a conserved feature for this family of toxins (Satchell 2007). *Vibrio cholerae* MARTX toxin is secreted by an atypical type 1 secretion system (T1SS). This T1SS is composed of four-component proteins: an ATP-binding cassette (ABC) transporter RtxB, a membrane fusion protein RtxD, an additional ATP-binding protein RtxE, and a TolC-like protein (Boardman and Satchell 2004; Linhartova et al. 2010). In *V. vulnificus*, a gene cluster next to the *rtxA1* operon encodes a high homolog of the MARTX-specific T1SS of *V. cholerae*. An *rtxE* gene in this cluster was mutated, and the results suggested that RtxE is essential for the virulence of this bacterium (Lee et al. 2008b). The disruption of the *rtxE* gene also blocked secretion of RtxA1 to the cell exterior and resulted in a significant reduction in cytotoxic activity against epithelial cells. As with the *rtxA1* mutant, infections of human epithelial cells with the *rtxE* mutant also cause lower levels of the poly(ADP-ribose) polymerase (PARP), cytochrome c, caspase-3, and mitochondrial membrane depolarization underscoring that *V. vulnificus* RtxA1 is secreted into the cell exterior through the T1SS (Lee et al. 2008b). In *V. cholerae*, MARTX is growth phase dependent. The toxin activity is detectable only in the supernatant fluid from log phase cultures. One of the regulation mechanisms is that exported proteases that destroy the activity of the secreted MARTXvc are expressed in the stationary phase. In addition, the expression of the T1SS gene is also under growth phase control, and it is repressed in stationary phase by a mechanism not linked to quorum sensing (Boardman et al. 2007). The MARTXvc toxin gene itself is also repressed in stationary phase; however, the mechanism of gene suppression remains to be unveiled. To date, there are no reports about the growth phase control of the RtxA1 toxin in *V. vulnificus*.

### Comparison of the action of *V. vulnificus* RtxA1 and *V. cholerae* MARTX

The RtxA1 toxin of *V. vulnificus* is overwhelmingly cytotoxic to host cells, whereas MARTXvc has much lower cytotoxic activity to host cells. In addition, contact-dependent acute cytotoxicity is induced by RtxA1 of *V. vulnificus* but not by *V. cholerae* MARTX. Another major difference between the action of the RtxA1 toxin of *V. vulnificus* and MARTXvc is that the latter induces covalent actin cross-linking, whereas the *V. vulnificus* toxin induces the actin aggregation but without these covalent cross-linking (Kim et al. 2008). The region of the MARTXvc toxin responsible for cross-linking activity is the actin cross-linking domain (ACD) (Sheahan et al. 2004). ACD has been demonstrated to act as an enzyme that directly binds actin and introduces the covalent cross-links (Cordero et al. 2006). ACD can cause depolymerization of actin stress fibers and an increase in paracellular permeability thus resulting in cell rounding (Cordero et al. 2006). RtxA1 of *V. vulnificus* has also been demonstrated to induce rapid cell rounding and to form plasma membrane blebs, while host cells showed noncovalent actin cross-linking (Kim et al. 2008). It is tempting to speculate that the different behaviors between the two toxins are due to the absence of the ACD domain in the *V. vulnificus* RtxA1. In MARTXvc, a region near the ACD is related to a small Rho GTPase inactivating activity. Rho GTPase inactivation domain (RID) also plays a role in cell rounding by MARTXvc (Sheahan and Satchell 2007). *V. vulnificus* RtxA1 contains a conserved RID region showing high homology with the *V. cholerae* MARTX. It was demonstrated that RtxA1 of *V. vulnificus* caused a rearrangement of F/G actin dynamics implying that RtxA1 might have substantial Rho inactivating activity responsible for the cell rounding phenotype (Kim et al. 2008). MARTX of *V. cholerae* was reported not to form cytoplasmic pores in host cells (Fullner and Mekalanos 2000). However, RtxA1 of *V. vulnificus* causes hemolysis of human RBC by forming pores at the cell membrane. Two unique domains of *V. vulnificus* compared with *V. cholerae* MARTX were suggested to play important roles for this function (Kim et al. 2008).

### The RtxA1 domain organization

As mentioned in the previous section, there are other two homologs of the *rtxA1* genes in the chromosome of *V. vulnificus* (Chen et al. 2003). In CMCP6 strain, one homolog, VV12715, is located in chromosome 1, and another homolog, VV21514, is in chromosome 2. These two homolog proteins have low similarities with RtxA1 in sequence, and none of them are associated with cytotoxicity or regulated by HlyU (Liu et al. 2007; Kim et al. 2008). Compared with MARTXvc, RtxA1 shares the N- and C-terminal repetitive regions, a Rho-GTPase inactivation domain (RID) which accounts for the cell rounding and Rho inactivation, an autocatalytic cysteine protease domain (CPD) which enables the toxin to undergo proteolytic cleavage during translocation into host cells, and an α/β-hydrolase domain (Roig et al. 2011). The difference between these two toxins is that *V. vulnificus* RtxA1 lacks an ACD and presents three additional putative domains, DUFs (domain of unknown function), Mcf (a domain similar to an Mcf toxin of *Photorhabdus luminescens*), and PMT C1/C2 (a domain similar to a portion of the *Pasteurella mitogenic* toxin PMT) (Roig et al. 2011). Analysis of the RtxA1 toxin domain organization in all three biotypes of *V. vulnificus* strains identified three different types of RTX toxins I, II, and III (Roig et al. 2011). All the three types of toxin have in common: CPD, α/β-hydrolase domain, and a domain resembling that of the LifA protein of *Escherichia coli* O127:H6 E2348/69 (Efa/LifA). *Vibrio vulnificus* biotype 1 strains harbor type I and II toxins, whereas biotype 2 strains carry the type III and biotype 3 strains encode the type II toxin (Roig et al. 2011). In a recent study, 40 *V. vulnificus* strains were examined and divided into four distinct variants of the *rtxA1* gene based on the different arrangements of the effector domains (Kwak et al. 2011). These variants might have resulted from recombination either with *rtxA1* genes carried on plasmids or with the *rtxA* gene of *V. anguillarum*. The most common *rtxA1* variant from clinical isolates of *V. vulnificus* encodes a toxin that has reduced potency and is different from the toxin produced by environmental strains suggesting that the *rtxA1* gene is undergoing significant genetic rearrangements.

## Another Player, the Regulator HlyU

Kim et al. used an in vivo induced antigen technology (IVIAT) and identified genes expressed during an actual human infection rather than in an animal model (Kim et al. 2003b). Several novel genes were identified that likely play important roles in the survival and replication of *V. vulnificus* in humans presenting a cirrhotic syndrome, with underlying conditions of iron excess. HlyU, a homolog of the *V. cholerae* regulator of the hemolysin gene, is one of the in vivo–induced genes. A *V. vulnificus hlyU* mutant nearly abolished cytotoxic activity. Furthermore, the mutant shows less virulence than the wild type (Kim et al. 2003b). It has been reported that the HlyU protein of *V. vulnificus* regulates the expression of the *vvhA* and *vvpE* genes encoding, respectively, a hemolysin/cytolysin and an elastolytic protease gene (Lee et al. 2004b). However, it was puzzling that neither the *vvhA* nor the *vvpE* mutant showed a decrease in virulence (Wright and Morris 1991; Shao and Hor 2000; Fan et al. 2001). Thus, it was clear that HlyU must have influence in the expression of other genes to explain the dramatic reduction in virulence in the *hlyU* mutant. Using microarray analysis, we reported that HlyU positively regulates the expression of the *rtxA1* operon genes (Liu et al. 2007). As the pioneering studies on HlyU were carried out in *V. cholerae*, in this review we will first give a brief account of the history of HlyU in this bacterium.

### The HlyU protein in *V. cholerae*

**Figure 1:**
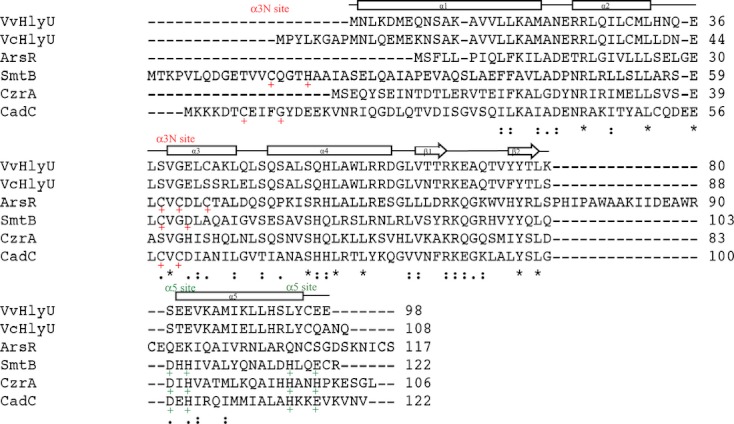
Sequence alignment of HlyU with representative members of the ArsR/SmtB family. VvHlyU:HlyU from *Vibrio vulnificus* CMCP6; VcHlyU:HlyU from *V. cholerae* N16961; ArsR is from *Escherichia coli* HS; SmtB is from *Synechococcus* PCC7942; CzrA is from *Staphylococcus aureus*; CadC is from *S. aureus* pI258. The HlyU protein similarity between *V. vulnificus* and *V. cholerae* is 93%, and the identity is 82%. Helices 2, 3, and 4 comprise a helix-turn-helix motif. α-Helices are shown as boxes and β-strands are shown as arrows. Identical residues are denoted by asterisk (*), whereas a colon (:) indicates conserved residues and a period (.) semiconserved residues. Metal-binding sites α3N and α5 are marked with plus (+) sign in red and green colors, respectively.

HlyU belongs to a family of small metal-regulatory transcriptional repressors including SmtB of *Synechococcus* sp. (Morby et al. 1993), ArsR of *E. coli* pR773 (Shi et al. 1994), CzrA of *Staphylococcus aureus* (Lee et al. 2006), and CadC of *S. aureus* pI258 (Endo and Silver 1995). Members of this family are “metal-sensor” proteins that normally repress the expression of operons association with concentrations of heavy metal ions that induce stress, while derepression results from direct binding of metal ions by these “metal-sensor” proteins. It has been reported that SmtB can sense zinc-ion (Huckle et al. 1993), ArsR senses As(III), Sb(II), and Bi (III) (Wu and Rosen 1993; Zhang et al. 2009), CzrA senses Zn (II) and Co(II) (Arunkumar et al. 2007, 2009), while CadC is a repressor that can sense Cd(II), Pb(II), or Zn(II) (Endo and Silver 1995; Sun et al. 2001; Busenlehner et al. 2002). Comparative structural and spectroscopic studies of several SmtB/ArsR family members revealed that these proteins harbor one or both of the two structurally distinct metal-binding sites, α3N or α5 (Fig. 1) (Busenlehner et al. 2002, 2003).

The *V. cholerae* HlyU protein is a 12.3-kDa protein containing a putative helix-turn-helix motif (Williams et al. 1993; Saha et al. 2006). Circular dichroism (CD) analysis showed that the *V. cholerae* HlyU protein is predominantly alpha helical (Saha et al. 2006). The *in silico* modeled structure of *V. cholerae* HlyU shows that it does not have the key metal-sensing residues like other members of this family. It is thus possible that HlyU evolved from an ancestral transcriptional repressor by loss of the metal-binding sites and is the only member of this family that has a positive regulatory function (Saha et al. 2006). In *V. cholerae,* HlyU regulates expression of the hemolysin encoded by the *hlyA* gene that is a virulence factor for this bacterium (Stoebner and Payne 1988; Williams and Manning 1991; Olivier et al. 2007). An *hlyU* mutant exhibits an increased LD_50_ in the infant mouse cholera model (Williams et al. 1993). Furthermore, the *hlyU* mutant, but not the *hlyA* mutant, is slightly defective in colonization competition assays, suggesting the possibility that the HlyU protein in *V. cholerae* regulates other factors in addition to hemolysin that might be critical for growth or colonization (Williams et al. 1993).

It was found that in *V. cholerae*, coordinately with *hlyA*, HlyU also regulates the expression of a 28-kDa secreted protein, Hcp (hemolysin-coregulated protein) (Williams et al. 1996). Two *hcp* genes were identified in *V. cholerae* and these 2 genes encode identical amino acid sequences and both express a 28-kDa protein. In non-O1, non-O139 *V. cholerae* strain V52, proteins Hcp-1 and Hcp-2 are secreted by a type VI secretion system (T6SS) and these two proteins are also required for the extracellular secretion of other T6SS substrates like VgrG-1 and VgrG-2 (Pukatzki et al. 2006). So far, the *hcp* gene is the only T6SS gene reported being regulated by the HlyU protein.

Although both genes *hcp* and *hlyA* are coregulated by HlyU, there are no obvious similarities between their promoters (Williams et al. 1996). This suggests the involvement of an intermediate regulator in the activation of *hcp* by HlyU, raising the possibility that HlyU is part of a regulatory cascade (Williams et al. 1996). Furthermore, although it is clear that HlyU is the positive regulator of the *V. cholerae hlyA* gene, there is no evidence indicating that HlyU interacts with the *hlyA* promoter sequences. Considering the situation in *V. vulnificus* that we will describe in the next sections it is possible that HlyU does not act as a direct transcriptional activator of *hlyA* but it interferes with a putative *hlyA* repressor.

### The HlyU protein in *V. vulnificus*

The *V. vulnificus* HlyU protein is an 11.9-kD protein. As shown in Figure 1, it is very closely homologous to the *V. cholerae* HlyU protein (93% of similarity and 82% identity) and less homologous to other proteins of this family. Like other members of this family, *V. vulnificus* HlyU contains a helix-turn-helix motif as evidenced by analysis of the solved HlyU crystal structure (Nishi et al. 2010). This typical winged helix-turn-helix (wHTH) motif is composed of three α-helices (α2, α3, and α4) and two β-strands (β1 and β2) as the wing. Like other proteins in the same family, HlyU of *V. vulnificus* forms a homodimer in the crystal structure and our recent bacterial 2 hybrid and native blue polyacrylamide gel results also suggested that HlyU exhibits a dimer (Nishi et al. 2010; Liu et al. 2011). Similar to HlyUvc, HlyU of *V. vulnificus* lacks metal-binding sites. The ligand residues cystine and asparagine in the first metal-binding motif are substituted with the serine and glutamine in HlyU, while the second metal-binding motif is not conserved at all in this protein (Fig. 1) (Nishi et al. 2010).

As previously mentioned, HlyU of *V. vulnificus* regulates the expression of the hemolysin/cytolysin gene *vvhA* and the elastolytic protease gene *vvpE* (Kim et al. 2003b). However, the *vvhA* mutant did not show a decrease in virulence by intraperitoneal infection using either an iron-normal or iron-overloaded mouse model (Wright and Morris 1991; Fan et al. 2001). In addition, the inactivation of the *vvpE* gene did not affect the ability of bacteria to infect mice and cause organ damage which indicates that HlyU must regulate the expression of other virulence-related gene(s) (Jeong et al. 2000; Shao and Hor 2000; Fan et al. 2001). We compared the transcriptome profiles of the *hlyU* mutant and the wild type *V. vulnificus* strain. The microarray data showed that in addition to a *toxT*-like gene cluster (our unpublished data), the *rtxA1* gene cluster was significantly downregulated in the *hlyU* mutant. In this cluster, other two genes VV20481 and VV20480 encode a predicted peptide chain release factor 1 and a predicted hemolysin acyltransferase, respectively (Lin et al. 1999). The mechanism of HlyU regulation on the *V. vulnificus rtxA1* operon was further analyzed using *lacZ* fusions. Here, we have introduced the two important role players in the virulence of *V. vulnificus*, HlyU and RtxA1. In the following section, we will discuss the mechanism of regulation of *rtxA1* in both *V. vulnificus* and *V. cholerae*.

## Mechanism of Regulation of the *rtxA1* Operon in *V. vulnificus* by the HlyU Protein

### HlyU is a derepressor of H-NS on the regulation of *rtxA1*

From the previous sections, it is clear that HlyU regulates the expression of the *rtxA1* operon in what appears to be a positive fashion, but what is the mechanism of this regulation? Our laboratory demonstrated that regulation by HlyU requires direct contact with a promoter located upstream of the *rtxA1* operon (−260 to −523) (Liu et al. 2007, 2009). The nucleotide sequence bound by HlyU was determined using DNase I foot-printing: TGTAATTATTAGTTTTTGTTAAATTAGCATTTTCTTTAAATT (between 376 and 417 bp upstream of the *rtxA1* operon) (Liu et al. 2007). This HlyU-binding site is very AT rich and contains an imperfect palindromic sequence suggesting that a dimer rather than a monomer of HlyU binds to this region.

**Figure 2:**
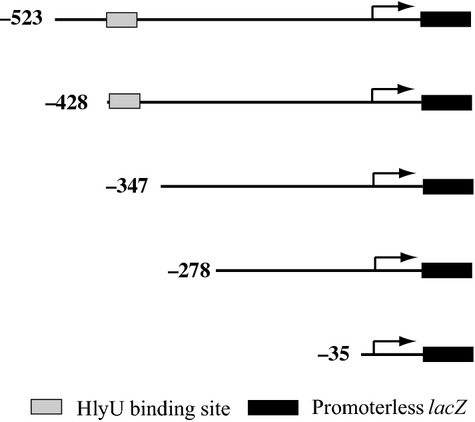
Scheme of various promoter-*lacZ* transcriptional fusions in the *hlyU*^+^ (Δ*lacZ*) and *hlyU*^−^ (Δ*lacZ* Δ*hlyU*) strains. The figures described the promoter fragments that were, respectively, fused to the *lacZ* gene.

How does the interaction of HlyU at this distal region from the promoter results in the enhancement of the transcription of the *rtxA1* operon? We constructed nested deletions of the promoter upstream region, fused them to the *lacZ* gene, as shown in Figure 2, and then assessed the expression of each one of them (Liu et al. 2009). An unexpected finding was that progressive deletions toward the −35 were concomitant with an increase in the basal transcription of the *rtxA1* promoter. The fusion containing an upstream endpoint at position −428 that still contained the HlyU-binding site became independent of the presence of the HlyU protein. Thus, it was obvious that HlyU might not be a positive regulator but operated by relieving the action of a repressor that must bind to a region near the HlyU-binding site. Searching the literature, it was clear that a good candidate for this repressor was the histone-like nucleoid structuring protein (H-NS) protein as it is a known gene repressor in many bacteria (Tendeng and Bertin 2003; Dorman 2004; Dorman and Kane 2009). Mutation of the gene encoding the H-NS protein indeed resulted in an increase of 15.5-fold in the transcription level of *rtxA1*. The expression of the *rtxA1* operon promoter fused to *lacZ* was also assessed under *hns*^+^ and *hns*^−^ backgrounds (Liu et al. 2009, 2009). The results indicated that regulation of the *rtxA1* operon by HlyU only existed in the *hns*^+^ rather than the *hns*^−^ background confirming that HlyU relieves the repression of the *rtxA1* operon genes caused by H-NS or a H-NS-associated factor. It is clear, therefore, that HlyU acts as a derepressor of H-NS in *V. vulnificus*. Using DNA footprint assays, we determined that two of the H-NS-binding sites, II and III, upstream of the *rtxA1* operon promoter, overlap with the HlyU-binding site (Liu et al. 2009).

**Figure 3:**
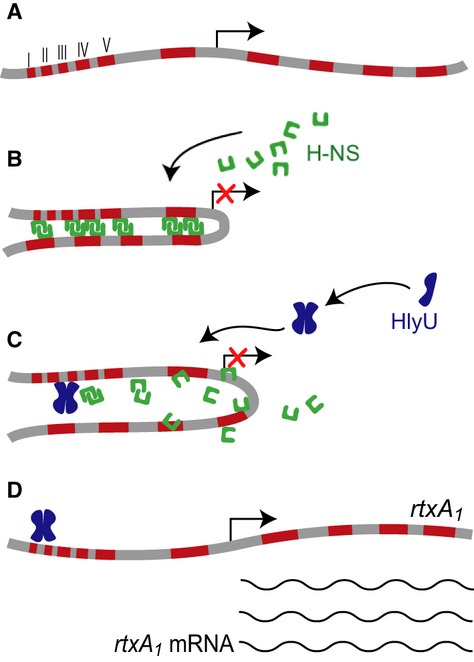
Model of the mechanism of HlyU regulation of the *rtxA1* toxin gene. (A) Upstream region DNA of the *rtxA1* operon. The arrow indicates the transcription start site of the *rtxA1* operon and the red boxes stand for the H-NS protein-binding sites I (−459 to −430), II (−422 to −392), III (−389 to −362), IV (−353 to −328), and V (−322 to −289). (B) Under certain conditions, for example, prior to bacteria contacting the host cells, H-NS binds to multiple sites on the upstream regions of *rtxA1* operon and forms a DNA:H-NS:DNA bridge to impede the movement or exclude the entry of RNA polymerase leading to the repression of *rtxA1*. (C) Under conditions where invasion occurs, HlyU outcompetes H-NS, and then binds to the upstream region of the promoter that is overlapped with H-NS-binding sites II and III, thus interfering the H-NS interaction with the DNA. (D) The DNA:H-NS:DNA bridge is disrupted and the transcription of the *rtxA1* operon genes starts.

What is the mechanism of this derepression at the distal *rtxA1* operon promoter? It is possible that the HlyU protein displaces the H-NS protein not only from the HlyU-binding sites but also from all the other H-NS-binding sites. Using the competitive DNase I foot-printing protection assays, we demonstrated that HlyU could displace H-NS from all the binding sites at a low HlyU concentration; however, H-NS needs a higher concentration to displace the bound HlyU-DNA (Liu et al. 2009). We further demonstrated that H-NS binds to DNA extending downstream of the promoter, suggesting that its binding may form a DNA:H-NS:DNA bridge structure, which has been proposed to trap the RNA polymerase at promoters (Dame et al. 2005; Dorman 2007; Lithgow et al. 2007). Based on all the experimental information gathered so far, here we propose a model (Fig. 3) of the derepression mechanism of the *rtxA1* operon genes by HlyU: at first, prior to the bacterium contacting the host cells, H-NS binds to multiple AT-rich upstream and downstream regions of the *rtxA1* operon promoter. The H-NS binding causes the DNA molecule to bend forming a DNA:H-NS:DNA bridge that either impedes the movement of RNA polymerase or excludes the entry of this enzyme thus repressing the expression of the *rtxA1* operon. Once the bacterium is ingested or invades from open wounds, the bacterium contacts the host cells and somehow the expression of the HlyU protein is induced. HlyU binds to the upstream region of the *rtxA1* promoter and replaces some of the H-NS molecules interfering and breaking the DNA:H-NS:DNA structure, resulting in *rtxA1* gene expression. In the next section, we will discuss the possible signals and/or conditions that induce *hlyU* expression.

### A regulatory network in the *rtxA1* gene expression

**Figure 4:**
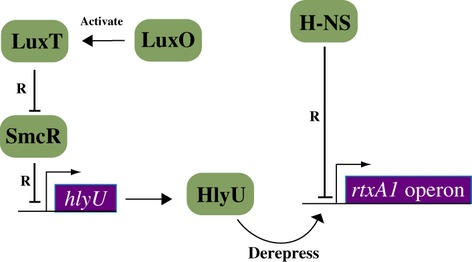
A regulatory cascade in the expression of the *rtxA1* gene of *Vibrio vulnificus*. LuxO activates the production of LuxT. LuxT protein represses the production of SmcR. Under conditions in which *smcR* is expressed, SmcR represses *hlyU* transcription via direct binding to the upstream region of the *hlyU* gene. It is additionally shown in this model that H-NS represses expression of the *rtxA1* operon by direct binding to the upstream region, and that HlyU binds to an overlapping region to replace H-NS from its binding site thus relieving the repression of *rxA1*. R represents “represses.”

A mutation of the LuxS quorum-sensing system causes a decrease in cytotoxic activity and an increase in transcription for the cytolysin/hemolysin gene *vvhA* in *V. vulnificus* suggesting that quorum sensing plays a role in the regulation of cytotoxicity in this bacterium (Kim et al. 2003a). Additionally, it was demonstrated that SmcR, a homolog of *V. harveyi* quorum-sensing regulator LuxR in *V. vulnificus*, binds to a region upstream of *hlyU* in *V. vulnificus,* acting as a repressor of *hlyU* expression (Shao et al. 2011). SmcR downregulates cytotoxicity and cytolysin/hemolysin production. Cytotoxicity downregulated by SmcR is attributed to both RtxA1 and VvhA, whereas a *ΔsmcR ΔhlyU* double mutant regained both cytotoxicity and cytolysin/hemolysin activity as long as the H-NS gene was also deleted (Shao et al. 2011). Thus, from these experiments, it is possible to deduce that SmcR mediates the regulation of cytotoxicity, and possibly virulence, by quorum-sensing signaling in *V. vulnificus* by repressing *hlyU*, the activator of *rtxA1* and *vvhA*. However, under normal physiological conditions, SmcR is repressed by the LuxT protein and the transcription of *luxT* is activated by the quorum-sensing regulator LuxO, and thus, H-NS becomes the sole controlling element that HlyU must contend with (Roh et al. 2006). The model in Figure 4 shows a plausible mechanism of how this may occur. However, an opposite finding on the roles of SmcR in cytotoxicity has also been reported (Lee et al. 2007b). It was found that a disruption of SmcR in the *V. vulnificus* strain ATCC29307 resulted in decreased cytotoxic activity to the human intestinal epithelial cell INT407. This discrepancy could be due to the different bacterial strains and/or cell lines used, and it is clear that more experiments need to be carried out to confirm these findings using multiple bacterial strains and cell types.

## The Regulation of Cytolysin/Hemolysin by HlyU in *Vibrios*

As mentioned in the previous sections, the HlyU protein also regulates production of the cytolysin/hemolysin VvhA in *V. vulnificus*. The role of the cytolysin/hemolysin in virulence has been controversial as the mutation of the *vvhA* gene did not affect the LD_50_ in mouse (Wright and Morris 1991; Fan et al. 2001). However, injection of the cytolytic toxin resulted in severe tissue damage and even death in the mouse model (Gray and Kreger 1987). Furthermore, VvhA exhibits minor cytotoxicity that is detectable only when the major cytotoxic factor RtxA1 is mutated (Kim et al. 2008). More recently, it was shown that VvhA plays an important role in the gut to promote early in vivo growth and dissemination of *V. vulnificus* from the small intestine to other organs adding new information to better understand the potential role of *vvhA* in the pathogenesis of *V. vulnificus* infection (Jeong and Satchell 2012). As for the regulation mode of *vvhA*, as explained above, it is clear that SmcR also negatively regulates the expression of *vvhA* by repressing *hlyU*. The cytolysin/hemolysin activity of Δ*hns*, Δ*hlyU* Δ*hns*, or Δ*smcR* Δ*hlyU* Δ*hns* mutants is significantly higher than their *hns*^+^ parent strains suggesting that H-NS also plays a negative regulation role on *vvhA* expression (Shao et al. 2011). Previously, it was reported that iron plays an essential role in the regulation of *vvhA* as *vvhA* transcription was repressed by iron via the Fur protein (Kim et al. 2009). However, under high iron concentration, the extracellular secretion of VvhA was increased as iron increases the activity of the PilD-mediated type II secretion system that is responsible for the hemolysin secretion (Kim et al. 2009).

In another *Vibrio* species, *V. anguillarum*, it was reported that HlyU also positively regulated the production of hemolysin (Vah1) and MARTX toxin (RtxA) that are considered virulence factors in this bacterium (Rock and Nelson 2006; Li et al. 2008). It was shown that HlyU directly binds to the promoters of *vah1* and the *rtxA* operon; however, it is still not known whether HlyU also functions as a derepressor antagonizing H-NS in this bacterium.

## Conclusions

The studies discussed in this work demonstrated that the *rtxA1* gene, encoding the repeat-in-toxin protein, is an important virulence factor of *V. vulnificus* and that the *rtxA1* gene is regulated by the HlyU protein. In its normal state, *rtxA1* expression is repressed by the H-NS repressor, and HlyU acts as a derepressor by binding to a region upstream of the *rtxA1* operon promoter resulting in the removal of the H-NS protein allowing *rtxA1* expression and bacterial invasion to occur (Liu et al. 2009). Further regulatory controls are related to the quorum-sensing genes *luxO* and *smcR*, thus suggesting that environmental parameters could also play a role in this regulation.

Comparing and contrasting the properties of HlyU from *V. vulnificus* and *V. cholerae*, it is clear that regulation by HlyU in these two bacteria is different. Furthermore, nearly all *V. cholerae* strains produce MARTX toxin; however, it does not play a predominant role in lethality and its role in intestinal disease is currently unclear (Olivier et al. 2007; Satchell 2007). A more thorough analysis of the role played by HlyU in *V. cholerae* will likely shed light on the intricacies of the mechanisms of regulation by HlyU.

## Obituary: Jorge H. Crosa

This article was submitted on 30th April by the corresponding author, Jorge H. Crosa, of the Oregon Health and Science University (OHSU). A few days later on 19th May Jorge suddenly deceased, leaving anyone who had the honor and pleasure to know him in disarray. Jorge was born in Buenos Aires and obtained his Ph.D. in Chemistry at the University of Buenos Aires. Together with his wife, Lidia, he immigrated to the United States in 1972 to work as a postdoctoral fellow in Washington. Later he worked with Dr. Stanley Falkow at the University of Washington in Seattle, and in 1981 he became an Assistant Professor at OHSU in the department of microbiology. His main field of investigation was on iron transportation and virulence of Vibrios, in which he obtained worldwide recognition. He published 145 articles and he was the recipient of several awards, including the prestigious Germany Humboldt Foundation Prize and the International Igor Stojiljkovic Memorial Award. He was an editor of *FEMS Microbiology Letters* and *Biometals*, and was on the editorial board of the ASM journals: *Journal of Bacteriology* and *Infection and Immunity*. He was a member of the NIH Study Sections on Microbial Physiology and Genetics, and on Bacterial Pathogenesis. Dr. Crosa was an elected fellow of the American Academy of Microbiology and President-Elect of the International Biometals Society. I met Jorge on several occasions at International Biometals meetings (he organized the 2006 edition in Portland). At the 2008 Biometals meeting in Santiago de Compostella (Spain), I remember that he asked me to organize the 2012 meeting, an offer I accepted because I liked him a lot. At the end of the meeting in Brussels, I presented a eulogy and everyone in the audience was moved. We will miss you Jorge – you were not only a top microbiologist, but a fantastic and friendly human being.
